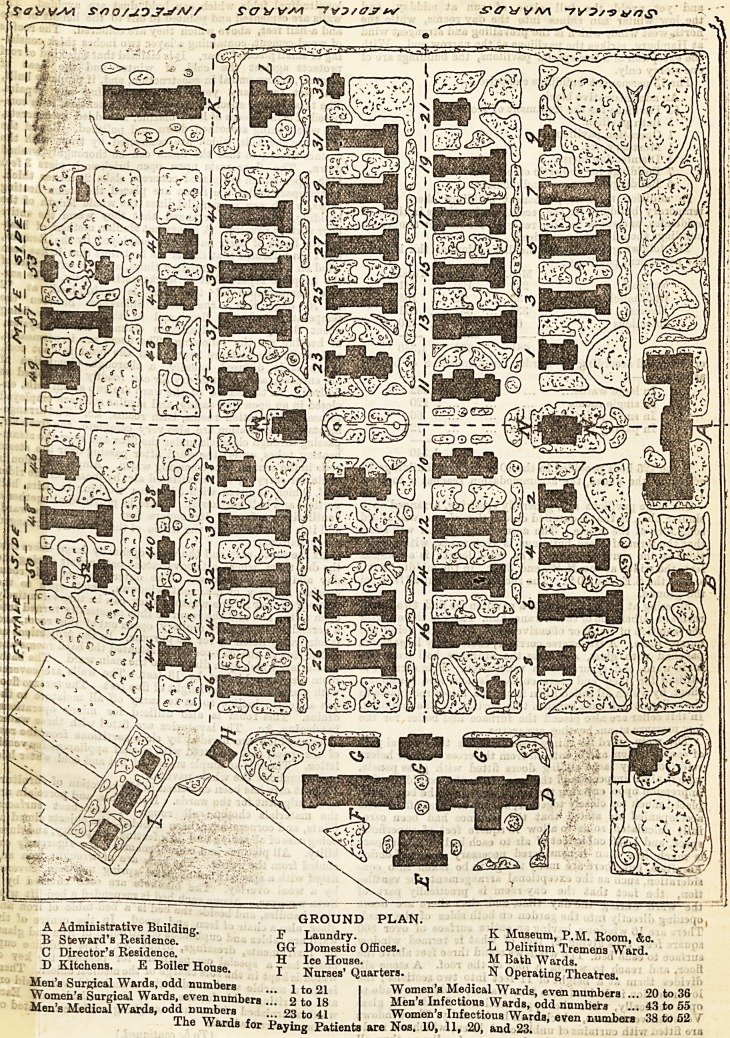# The New General Hospital at Eppendorf Hamburg

**Published:** 1891-11-28

**Authors:** Conrad W. Thies

**Affiliations:** Secretary to the Royal Free Hospital


### A Model Hospital.

By Conrad W. Thies, Secretary to the Royal Free Hospital.

### THE NEW GENERAL HOSPITAL AT EPPENDORF, HAMBURG.

Hamburg may justly claim a foremost place amongst the commercial cities of Europe, and the most casual visitor could hardly fail to observe its exceptional advantages, not only as a great business centre, but also as a desirable place of residence. But to members of the Hospitals Association, and indeed, to all who are specially interested in hospitals and their work, its principal attraction will probably be that it possesses a modernly constructed hospital, which our present Chairman, Mr. Jonathan Hutchinson, has aptly described as "the best example of elaborate arrangements for the restoration of health which yet exists" (*Archives of Surgery*). Such high praise from so eminent an authority naturally stimulated my desire to visit this institution, and during a recent holiday tour through North Germany I availed myself of the opportunity of visiting the New Hospital of Hamburg, which, in many respects, was a revelation to me of what such an institution ought to be.

Prior to 1885 Hamburg already possessed a large general hospital, built in 1820, with accommodation for 1,300 patients, which was increased by extensions at various times to 1,600 beds, but for some years this provision for the medical needs of the rapidly-increasing population was found to be quite inadequate, and the Municipal Council, after considering every possible expedient for further enlargement of the existing institution, resolved to build an entirely new hospital for the reception of the more acute and serious cases, utilising the old hospital for the treatment of out-patients, and for chronic and infirm cases. This important decision was hastened by the fact that the old hospital was already dangerously overcrowded, nearly 2,000 patients being under treatment therein, and also by the presence of cholera in the South of Europe.

Having thus decided, the municipality selected Herr Zimmerman, the architect, and Dr. Curschmann, the director of the old hospital, to whom they gave almost absolute discretion, in respect to the choice of site, system of construction, and general hygienic arrangements. A suitable site was selected at Eppendorf, a suburb two and a-half miles from the heart of the city. It comprised 45 acres, situated upon high and undulating ground in the midst of fields and nursery gardens, the nearest buildings being fully a quarter of a mile distant. The soil was sandy with a thin covering of mould, and the land had never previously been built upon. Hamburg and its suburbs are covered by a complete network of tramways, and it is part of the scheme to carry the tram lines into the grounds of the new hospital, and to construct special ambulance tramcars so as to facilitate the removal of patients from the old hospital, and all parts of the city and suburbs.

The work was commenced in 1885, and completed in 1889. As it was intended for the reception of acute and grave maladies requiring the very best hygienic conditions, it was, after most careful consideration, decided to construct the hospital upon the pavilion system, in preference to the corridor-pavilion plan, it being believed that the advantages of this system would far outweigh its disadvantages, and it is now claimed by the authorities that two years' experience has fully justified this decision.

A reference to the ground plan will show that the hospital comprises 83 separate buildings. Passing through the principal entrance in the centre of the administrative building, we find a broad paved pathway which divides the grounds into two equal portions, that on the right or N.E. side being reserved for the men, and that on the left or S.W. side for the women and children. This main path is intersected at right angles by six other paths which separate the various rows of pavilions. The first two rows are devoted to surgical, the second two to medical, and the remainder to infectious wards. The buildings are at least twenty feet apart, and the intervening spaces are laid out with grass lawns ornamented by flower beds and shrubs. No trees are permitted, as they would impede the free movements of air. I observed that many of the grass lawns were provided with tents and seats covered by awnings for the use of convalescent patients in fine weather. The kitchens and domestic offices are situated upon the highest portions of the ground, so that there is a gentle slope towards the pavilions, which facilitates the transport of provisions and other supplies. The administrative buildings and domestic offices, and the whole of the paths, are lighted by gas, but all the wards and rooms used by the patients are illuminated by electricity, which is generated in a special building. All the pavilions are in telephonic communication with the administrative building, which again is in direct telephonic communication with the old hospital, the police and fire stations, and also with the telephone system of the City and suburbs. There is an ample supply of hydrants and other appliances for the extinction of fire. The pavilions are each furnished with two large water tanks, one for supplying the baths, lavatories, &c., and the other, fitted with filtering apparatus, for drinking purposes. The drainage system is on the most approved modern principle, and is connected with the main sewers of Hamburg. There is a special building for disinfecting purposes, and here all products of typhoid, phthisis, and other infectious diseases are disinfected before being discharged into the drains.

The pavilions are plainly constructed of brickwork, relieved by glazed tiles and stone dressings. They are arranged with their long axis running from north-west to south-east. By this arrangement it is secured that both sides of each pavilion receive a share of sunlight,

GROUND PLAN.

and yet avoid the full glare of sun at midday, and the low winter sun shines into the day-rooms, while the north-west wind, which is the prevailing and strongest wind at Hamburg, strikes the pavilions at the narrow end. With the exception of a few special pavilions, the buildings are of one storey only.

The 73 permanent buildings comprise :—

An administrative block.
55 Pavilions for the treatment of the sick.
1 Bath pavilion.
1 Operation pavilion.
1 Mortuary, &c.
1 Disinfecting house.
7 Buildings for domestic offices, &c.
5 Buildings for nurses and officials.

In addition to these permanent buildings there are 10 temporary buildings for the reception of patients in times of epidemics, and space is also reserved for further temporary buildings should they be required.

The following table shows the disposition of the beds in the permanent pavilions :—

| | Men. | Women. | Totals. |
|---|---|---|---|
| Surgical | 251 | 190 | 441 |
| Ophthalmic | 36 | 72 | 108 |
| Medical | 334 | 337 | 671 |
| Infectious | 60 | 60 | 120 |
| | 681 | 659 | 1,340 |

or, arranged according to wards, the disposition of beds is as follows :—

| | |
|---|---|
| In large wards... | 1,082 |
| In single rooms | 120 |
| In small isolated wards | 66 |
| In paying patients' rooms | 72 |
| | 1,340 |

If the 126 beds provided in the 10 temporary buildings be added to the above, the accommodation reaches a total of 1,500 beds.

The large pavilions are, generally speaking, arranged on a similar plan, so that a detailed description of one will afford some idea of the whole. The accompanying plan will show that the form of these buildings is rectangular with projections at the south-east end. The total length is about 132 feet, the width at the N.W. end 39 ft., in the middle 31 ft., and at the S.E. end 60 ft. The roof stands about 20 ft. from the ground.

Entering at the N.W. end of a pavilion we find two small rooms on either side of the entrance hall. Three of these are for the isolation of noisy or offensive cases, the fourth being the nurses' room. A transverse corridor, enclosed by large doors panelled with glass, separates the entrance hall from the main ward, and secures cross ventilation. This corridor is utilised for linen and store cupboards, and also contains a shoot, by which all soiled linen is carried into a receptacle in the cellar, which runs beneath this portion of the building. In this cellar are also placed the furnace and boiler for the low pressure steam heating apparatus.

The main ward for thirty beds occupies the central part of each pavilion, and is entered from the cross corridor before referred to, by large sliding doors fitted with glass panels, which allow inspection of the ward from the corridor. My impression upon entering the ward was that the beds were placed much too closely together, but the medical officer who accompanied me stated that the distance had been carefully calculated, so as to allow 75 square feet of superficial space, and 1,250 cubic feet of air to each bed. This air space is much less than is considered necessary in our London hospitals, but there are many things to be taken into consideration, such as the exceptional arrangements for ventilation, the fact that the day-room is practically part of the ward during the day time, and that there are doors opening directly into the garden on both sides of the ward. There are 16 windows, having an entire surface of over 800 square feet, thus giving 27 feet of what is termed window surface to each bed. The windows stand three feet above the floor, and reach to within a foot of the roof. A stone sill divides them at a height of 11 feet into two sections ; the lower section is divided into four frames, which are made to open vertically, while the upper section is fitted with glass Venetian ventilators, which open horizontally. The windows are fitted with curtains of unbleached linen, which are made to draw across from the sides by means of pulleys ; thus all longitudinal folds which can hold dust are avoided. The walls are cemented and painted in oils to the height of five and a-half feet, above which they are coloured. The roof is of wood-cement, containing a layer two inches thick consisting of small piecesof spar. It is claimed that this kind of roof protects against the cold of winter and the heat of summer, is economical in construction, and, if properly built, will last for many years without needing any repairs. The terazzo floor specially attracted my attention, it is composed of small pieces of hard marble, imbedded in concrete, the whole being smoothed down and polished. Round the walls are channels, which are connected by traps with the surface drains, so that the floors can be thoroughly washed down. The floor rests upon open brickwork piers 3 feet high, upon which are placed concrete slabs 2½ inches thick. In the space under the floors are placed the steam heating tubes, which run in parallel lines the whole length of the building, and are so arranged that they warm the entire floor surface equally. A somewhat similar method of heating the floors of their baths was employed by the Romans, but I am informed that this is the first time that it has been tried on a large scale in modern times, and the authorities of the hospital report that it works satisfactorily. The surface temperature of the floor is maintained at 72 to 76 deg. Fah., the mean difference between the temperature of the floor and of the air above being about 9 deg. The advantages claimed for this system of heating are the following: The floors are impermeable, and while the feet are kept warm the head is kept cool, the heat is diffused equally, a thorough circulation of air ensured, and moist or dry air can readily be supplied as desired. In the centre of the ward are two steam coils, enclosed in an open iron casing, and similar coils are employed to heat the isolation rooms, bath rooms, &c. The arrangements for ventilation are also very complete, the fresh air, passing through channels under the floor and over the steam-heating tubes, enters the ward after passing over the steam coils in the centre, thus emerging warm. Ample provision is made for carrying off the vitiated air by means of the rider roof, which is fitted with 52 valves working in pairs, half vertical and half horizontal. There is provided altogether a ventilating surface of 168 square feet for the entire ward, and it is calculated that the whole of the air is thus changed every half-hour.

At the south-east end the ward opens by large doors into the day-room, where all convalescent patients sit during the day and take their meals. The outer wall of this room consists entirely of casement windows, which open to the ground, so that in fine weather they can be thrown entirely open to the air, and the room be thus practically turned into a verandah. The ward scullery opens out of this room, and is admirably fitted up for its purpose. In the projections on either side of the day-room are situated the bath rooms and lavatories, which open direct into the main ward The bath-room measures 14 feet by 13 feet. The walls are covered with glazed tiles to the height of five feet. The terazzo floor has a gentle incline so as to carry off all moisture to the drains. This room is also utilised for minor operations, bandaging, &c. The lavatories are fitted with three water-closets, which are divided by slate partitions from the rest of the space, and are fitted with special appliances for ventilation. There is an ample supply of hot and cold water laid on throughout the whole pavilion.

Great care has been exercised in the selection of furniture and equipment for the wards. Iron, glass, and porcelain are the materials chosen: all rough or absorbent surfaces, joints, and corners have been avoided, the objects aimed at being ease of cleansing and disinfecting, simplicity, and durability. All pictures and mere ornament are rigorously excluded from the wards. The beds are of wrought iron and fitted with a special kind of metal spring mattress, covered by a wool overlay. The blankets are each covered by a linen wrapper. Above each bed is suspended a bed-lift with glass handles, and beside the bed is a bed-table of iron and glass, also a chair of iron and wood. In the centre of the ward are tables and cupboards constructed of iron and glass, for instruments, dressings, drugs, &c. The medicine cupboard has a special compartment for poisons, the key of which is kept in the sole charge of the medical officer. There is also a marble washstand with hot and cold water laid on, and a writing table for the use of the medical staff. Each ward is furnished with a chair weighing machine mounted on rubber-covered wheels.

*(To be continued.)*

## Figures and Tables

**Figure f1:**